# Prediction of Acute Cardiac Rejection Based on Gene Expression Profiles

**DOI:** 10.3390/jpm14040410

**Published:** 2024-04-12

**Authors:** Bulat Abdrakhimov, Emmanuel Kayewa, Zhiwei Wang

**Affiliations:** 1 Department of Cardiovascular Surgery, Renmin Hospital of Wuhan University, Wuhan 430060, China; bulat@whu.edu.cn; 2 School of Computer Science, Wuhan University, Wuhan 430072, China; kayewa.emmanuel@gmail.com

**Keywords:** acute cardiac rejection, heart transplantation, machine learning

## Abstract

Acute cardiac rejection remains a significant challenge in the post-transplant period, necessitating meticulous monitoring and timely intervention to prevent graft failure. Thus, the goal of the present study was to identify novel biomarkers involved in acute cardiac rejection, paving the way for personalized diagnostic, preventive, and treatment strategies. A total of 809 differentially expressed genes were identified in the GSE150059 dataset. We intersected genes selected by analysis of variance, recursive feature elimination, least absolute shrinkage and selection operator, and random forest classifier to identify the most relevant genes involved in acute cardiac rejection. Thus, HCP5, KLRD1, GZMB, PLA1A, GNLY, and KLRB1 were used to train eight machine learning models: random forest, logistic regression, decision trees, support vector machines, gradient boosting machines, K-nearest neighbors, XGBoost, and neural networks. Models were trained, tested, and validated on the GSE150059 dataset (MMDx-based diagnosis of rejection). Eight algorithms achieved great performance in predicting acute cardiac rejection. However, all machine learning models demonstrated poor performance in two external validation sets that had rejection diagnosis based on histology: merged GSE2596 and GSE4470 dataset and GSE9377 dataset, thus highlighting differences between these two methods. According to SHAP and LIME, KLRD1 and HCP5 were the most impactful genes.

## 1. Introduction

Heart transplantation is a lifesaving intervention in the setting of end-stage heart disease, such as heart failure [1]. Despite gradual improvement in 1-year survival rates for cardiac transplantation, acute cardiac allograft rejection remains a significant challenge in the post-transplant period, necessitating meticulous monitoring and timely intervention to prevent graft failure [2,3]. Discovering biomarkers crucial in cardiac rejection may aid in the development of targeted therapies and improve heart transplantation outcomes. Allograft rejection involves both antibody and T cell responses [4]. Cytotoxic T lymphocytes and natural killer (NK) cells play a pivotal role in the immune response including in organ transplantation [5,6]. They contribute to allograft rejection by releasing perforin and granzymes as part of their cytotoxic mechanisms [7]. Numerous genes modulate antibody responses and T cell function, thereby impacting allograft rejection. For instance, genes encoding major histocompatibility complex (MHC) molecules, such as human leukocyte antigen (HLA) genes, play a central role in antigen presentation to T cells and are important determinants of graft survival [8,9]. Moreover, genes encoding co-stimulatory molecules (e.g., CD40) and adhesion molecules (e.g., ICAM-1) regulate T cell activation and migration [10,11]. Specific receptor genes, such as killer cell lectin-like receptors (KLRs) expressed on NK cells, can interact with MHC class I molecules on target cells to regulate NK cell activity and contribute to allograft rejection [12].

The advent of molecular diagnostics, namely the Molecular Microscope Diagnostic System^®^ (MMDx), has revolutionized the assessment of allograft rejection through comprehensive analysis of gene expression profiles in transplanted organs [13]. MMDx provides novel insights into the understanding of rejection states [14]. In parallel, machine learning algorithms present an incredibly powerful method to identify patterns from large, complex, and assorted data, such as gene expression data. Machine learning is widely used for predictive modeling in numerous fields and has been shown to outperform conventional statistical analysis tools in various settings [15,16]. Machine learning algorithms have shown enormous potential to open new frontiers with great prospects for personalized medicine [17]. By analyzing high-dimensional datasets, machine learning algorithms can identify molecular signatures indicative of rejection, which, in turn, may improve transplant outcomes by facilitating timely diagnosis and prompt treatment [18]. By integrating MMDx data with state-of-the-art machine learning algorithms, our objective was to develop a predictive model capable of accurately identifying acute rejection in heart transplant recipients. Moreover, through comprehensive analysis of gene expression profiles, we aimed to identify novel biomarkers and molecular pathways involved in cardiac rejection, paving the way for personalized diagnostic, preventive, and treatment strategies.

## 2. Materials and Methods

### 2.1. Selection Criteria

The gene expression omnibus (GEO) database was searched for “cardiac rejection OR heart rejection” from inception until 14 January 2024. Inclusion criteria were as follows: GEO series, expression profiling by array, human endomyocardial biopsy, datasets containing acute cardiac rejection and non-rejection samples, and at least 20 samples in a dataset. Five datasets were identified: GSE2596, GSE4470, GSE9377, GSE124897, and GSE150059. GSE124897 was excluded as all samples from it can be found in GSE150059.

GSE150059 (GPL16043 platform) contains 1320 samples: 853 with and 467 without acute cardiac rejection. The diagnosis of each sample within the GSE150059 dataset was based on MMDx, whereas histologic diagnosis was provided in GSE2596, GSE4470, and GSE9377. GSE2596 and GSE4470 share the same platform—GPL1053. The former dataset contains 63 samples (including 11 replicates): 35 stable samples and 21 rejected samples. Seven samples did not have a clear histologic diagnosis and were therefore excluded. GSE4470 contains 15 rejection and 12 non-rejection samples with one and two replicates, respectively. Finally, GSE9377 (GPL887 platform) consists of 9 stable and 17 rejected grafts. Detailed information regarding each dataset is provided in Table 1.

### 2.2. Data Preprocessing

**Figure 1:**
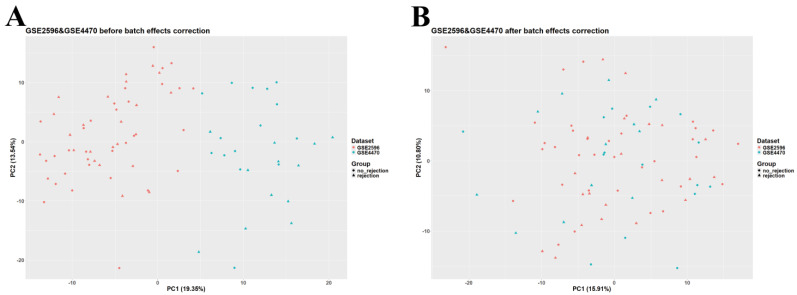
Principal component analysis (PCA) scatter plot of the merged dataset (GSE2596 and GSE4470). (**A**) Before adjusting for batch effects. (**B**) After adjusting for batch effects.

Data analysis and preprocessing were carried out in RStudio v2023.12.1 (R version 4.3.2, Bioconductor version 3.18). The *GEOquery* package (version 2.70.0) was utilized to download normalized GSE2596, GSE4470, GSE9377, and GSE150059 datasets. Given the large sample size of GSE150059 and the differences among datasets in terms of diagnostic methods, GSE150059 was selected as a discovery set and used for further analysis, whereas the other datasets were used as external validation sets. GSE2596 and GSE4470 were merged as they are biologically and technologically similar. *ggplot2* (version 3.4.4) was employed to construct a principal component analysis (PCA) plot to assess the presence of batch effects. The ‘removeBatchEffect’ function (available in the *limma* package, version 3.58.1) was used to adjust for batch effects in the merged dataset (Figure 1). The merged GSE2596 and GSE4470 dataset was used as the first external validation set, and GSE9377 was used as the second external validation set. Gene annotation in all datasets was carried out using information obtained from their corresponding platforms. Rows containing unspecific probes or probes not corresponding to any gene symbols were deleted; rows with duplicated gene symbols were merged, and a median was calculated.

### 2.3. Identification of Differentially Expressed Genes

*limma* was used to identify differentially expressed genes (DEGs). First, a linear model was built (‘lmFit’ function, default arguments), then the ‘eBayes’ function was employed to calculate empirical Bayes statistics (robust limma-trend method). Results were extracted using the ‘topTable’ function and were adjusted by the Benjamini–Hochberg procedure. The cutoff values were as follows: |logFC| > mean(logFC) + 2SD(logFC) and adjusted *p*-value < 0.05. Thus, DEGs with logFC > 0.4497 were considered upregulated, and DEGs with logFC < −0.4497 were considered downregulated. Volcano plots and heatmaps were created using the *EnhancedVolcano* (version 1.20.0) and *pheatmap* (version 1.0.12) packages.

### 2.4. Enrichment Aanalysis

*org.Hs.eg.db* (version 3.18.0) and *clusterProfiler* (version 4.10.0) were used to carry out Gene Ontology (GO) analysis to explore biological processes of upregulated and downregulated DEGs involved in acute cardiac allograft rejection. Kyoto Encyclopedia of Genes and Genomes (KEGG) enrichment analysis was also performed. *p*-value < 0.05 was considered statistically significant. *ggplot2* was utilized to construct the necessary plots.

### 2.5. Data Preprocessing for Machine Learning Analysis

Machine learning analysis was conducted in Python. GSE150059 was randomly split into a training set (70%), test set (15%), and internal validation set (15%). To ensure consistency in model training and validation, the features present in both the training and external validation datasets were aligned. This alignment guarantees that the models are trained and evaluated on an identical set of features, which is essential for accurate model performance assessment. Missing data points in datasets were addressed by employing Scikit-Learn’s *SimpleImputer* with a ‘median’ strategy to impute missing values. This approach ensures that the dataset is complete, allowing for effective model training and validation. The final preprocessing step involved scaling the features to have a mean of zero and a standard deviation of one, which was performed using Scikit-Learn’s *StandardScaler* transformer.

### 2.6. Feature Selection

In the pursuit of identifying candidate genes for heart transplant outcomes, various feature selection techniques were applied, each with its unique approach to isolating the most relevant features from the gene expression data. The following feature selection techniques were utilized:

1. Analysis of variance (ANOVA) was leveraged to pinpoint the top 100 genes with significant expression differences between conditions, using *SelectKBest* with the *f_classif* score function. This approach narrows down the feature space to those most impactful for the analysis;
2. Recursive feature elimination (RFE), through *RFECV*, combined with logistic regression and cross-validation (*StratifiedKFold*), dynamically identifies an optimal subset of features. Unlike traditional RFE which requires a predefined feature count, *RFECV* automatically determines the best number of features by maximizing cross-validation accuracy, making the selection process more data-driven;
3. The least absolute shrinkage and selection operator (LASSO), applied via *LassoCV*, optimizes feature selection alongside model training by identifying non-zero coefficient features through cross-validation. This method effectively reduces the feature set to those most predictive of outcomes without pre-specifying a feature count;
4. Random forest classifier (RFC) assesses feature importance after being trained with 50 trees. The optimal number of trees is found by using *GridSearchCV*. *SelectFromModel* with a ‘mean’ importance threshold is then used to filter the most significant features, allowing the model to concentrate on variables with the greatest impact on transplant outcomes.

### 2.7. Machine Learning Algorithms

Overlapping genes selected by feature selection tools were used to train the machine learning models. The models tested include logistic regression (LR), support vector machines (SVM), random forest (RF), gradient boosting machines (GBM), K-nearest neighbors (KNN), XGBoost, decision trees (DT), and neural networks (using the *MLPclassifier* in Scikit-Learn). Each model was evaluated on its ability to utilize the gene expression profiles for outcome prediction, with a focus on identifying the most effective model or combination of models. For each model, Scikit-Learn’s *GridSearchCV* was applied to explore a wide range of hyperparameters, identifying the combination that yields the best performance. The following metrics were used to provide insights into various aspects of model performance: accuracy, precision, recall (sensitivity), F1 score, Matthew’s correlation coefficient (MCC), area under the receiver operating characteristic curve (AUC), and area under the precision–recall curve (AUPRC) [19,20].

To ensure the generalizability and robustness of the models, a rigorous validation approach was employed, comprising both cross-validation and internal validation. Specifically, k-fold cross-validation was utilized, dividing the dataset into five smaller sets, training the model on four folds, and validating it on the remaining one, repetitively cycling through all folds.

### 2.8. Model Interpretation

To understand the impact of selected gene features on model predictions and the biological relevance of these genes in heart transplant outcomes, interpretation frameworks such as SHapley Additive exPlanations (SHAP) and Local Interpretable Model-agnostic Explanations (LIME) were used. SHAP provides insights into how each feature contributes to the model’s prediction for an individual sample, and LIME offers explanations for model predictions on individual instances, facilitating understanding of model behavior in specific cases.

## 3. Results

### 3.1. Identification of DEGs and Enrichment Analysis

After preprocessing, there were 19,042 genes in the GSE150059 dataset, 11,849 genes in the merged GSE2596 and GSE4470 dataset, and 16,546 genes in the GSE9377 dataset. As MMDx was used to diagnose acute cardiac rejection in GSE150059 and the rejection status of samples in the other datasets was based on histologic examination, GSE150059 was used to conduct differential expression analysis as well as GO and KEGG enrichment analyses, whereas the other datasets were used as external validation sets.

**Figure 2:**
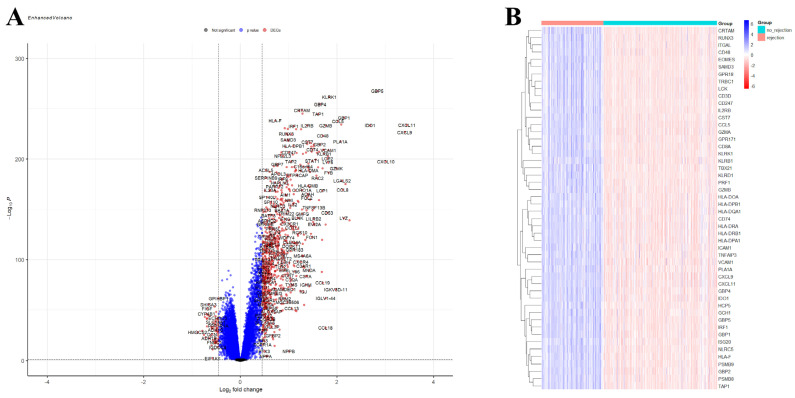
Differential expression analysis of GSE150059. (**A**) Volcano plot. Black dots—not significant genes, blue dots—genes with *p*-value < 0.05 but |logFC| < 0.4497, red dots—differentially expressed genes (*p*-value < 0.05, |logFC| > 0.4497). (**B**) Heatmap of the top 50 differentially expressed genes.

**Figure 3:**
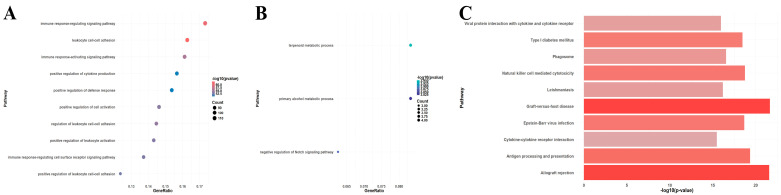
Enrichment analysis of differentially expressed genes in GSE150059 (top 10 pathways). (**A**) Gene Ontology (GO) enrichment analysis of upregulated genes. (**B**) GO enrichment analysis of downregulated genes. (**C**) Kyoto Encyclopedia of Genes and Genomes (KEGG) pathway enrichment analysis of all genes.

A total of 750 upregulated and 59 downregulated DEGs were identified in the GSE150059 dataset (Figure 2). Upregulated genes were mainly enriched in the immune-related biological processes, including immune response-regulating signaling pathway, leucocyte cell–cell adhesion, immune response-activating signaling pathway, etc. (Figure 3A). Downregulated genes were enriched in metabolic processes and cell signaling systems (Figure 3B). Notably, KEGG enrichment analysis revealed that DEGs were significantly enriched in graft-versus-host disease and allograft rejection (Figure 3C).

### 3.2. Machine Learning Analysis

**Figure 4:**
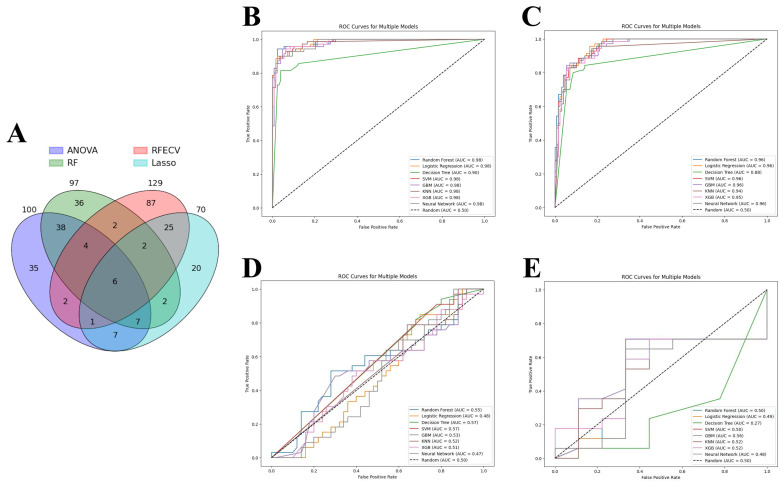
Machine learning analysis. (**A**) Venn diagram of genes selected by four feature selection tools. (**B**–**D**) Receiver operating characteristic (ROC) curve of machine learning models: (**B**) Test set; (**C**) Internal validation set; (**D**) External validation set 1 (merged GSE2596 and GSE4470). (**E**) External validation set 2 (GSE9377). Note: ANOVA—analysis of variance, RFE—recursive feature elimination, LASSO—least absolute shrinkage and selection operator, RF—random forest classifier; SVM—support vector machines, GBM—gradient boosting machines, KNN—K-nearest neighbors, XGB—XGBoost.

ANOVA, RFE, LASSO, and RFC were applied to identify the most relevant DEGs that contributed to the predictive power of machine learning models. As a result, 129 genes were selected by RFE, followed by 100 genes by ANOVA, 97 genes by RF, and 70 genes by LASSO. As each feature selection method has its own advantages and disadvantages, all selected genes were intersected to identify candidate genes involved in acute cardiac allograft rejection (Figure 4A). Thus, six genes were selected to train the machine learning models: HCP5, KLRD1, GZMB, PLA1A, GNLY, and KLRB1. Eight models showed similar performance when predicting acute cardiac rejection based on MMDx. According to aggregate metrics, RF and LR performed slightly better compared to the other models in the test and internal validation sets (Table 2 and Figure 4B,C). In contrast, DT underperformed compared to the other models and had an accuracy of 0.91 and AUC of 0.90 in the test set and an accuracy of 0.87 and AUC of 0.88 in the internal validation set.

Finally, we wanted to assess whether models trained on the MMDx dataset can be used to predict the histologic diagnosis of acute cardiac allograft rejection. LR, SVM, RF, GBM, KNN, XGBoost, DT, and neutral networks were tested on two external validation sets: merged GSE2596 and GSE4470 dataset and GSE9377 dataset (Table 3 and Figure 4D,E). All the models had very poor performance (close to random curve) in two external validation sets, highlighting major differences between the two diagnostic methods.

### 3.3. Model Interpretation

**Figure 5:**
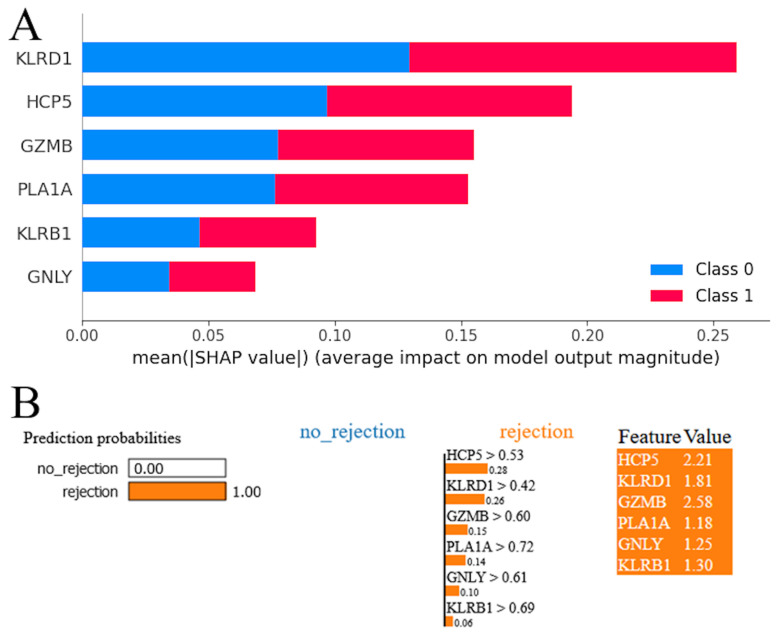
Model interpretation. (**A**) SHapley Additive exPlanations (SHAP) analysis for random forest model. (**B**) Local Interpretable Model-agnostic Explanations (LIME) interpretation for random forest model.

The SHAP summary plot for the RF model revealed a hierarchy of genes according to their influence on the model’s predictions (Figure 5A). Red and blue colors occupy half of the horizontal rectangles for each class. This means that each feature has an equal impact on the classification of both rejection and stable cases. The gene KLRD1 emerged as the most influential, exhibiting the highest mean impact on the model’s output. It was followed in significance by HCP5, suggesting that these two genes have a predominant role in the predictive framework. GZMB and PLA1A were also identified as impactful, albeit to a lesser extent than KLRD1 and HCP5, underscoring their contributory roles in the model’s decision-making process. KLRB1 and GNLY, while still influential, demonstrated a comparatively lower impact on the model’s predictions. The LIME plot indicates that the model predicts acute cardiac rejection with a probability of 1.00, suggesting strong confidence in this outcome (Figure 5B). All genes were shown with positive weights, indicating their influence on the acute cardiac rejection prediction. The values next to each feature represent their presence in the instance, and the weights (e.g., 0.28 for HCP5 > 0.53) show each gene’s contribution to pushing the prediction towards acute cardiac allograft rejection. High feature values alongside positive weights confirm their significant role in the model’s decision-making process for this prediction.

## 4. Discussion

We intersected genes selected by four robust feature selection methods (ANOVA, RFE, LASSO, and RFC) to identify the most relevant DEGs. HCP5, KLRD1, GZMB, PLA1A, GNLY, and KLRB1 were selected and were used to train the machine learning models. All DEGs, including the identified six genes, were mainly enriched in immune-related processes and pathways, including graft-versus-host disease and allograft rejection. These six genes have long been known to be associated with immunity and acute rejection [21,22,23,24,25,26,27,28,29,30,31,32]. HCP5 (HLA complex P5) is a long non-coding RNA. Its single-nucleotide polymorphisms were found to be associated with an increased risk of relapse, decreased survival rate, and occurrence of graft-versus-host disease in hematopoietic stem cell transplantation [21,22,23]. However, the role of HCP5 in solid graft rejection is unclear and requires further investigation. KLRD1 (CD94) and KLRB1 (CD161) are NK cell receptors involved in cytotoxicity and both antibody- and T-cell-mediated rejection [24,25,26]. CD94 forms a heterodimeric receptor with NKG2 isoforms resulting in either activating (e.g., CD94/NKG2D) or inhibitory (e.g., CD94/NKG2A) receptors, both of which bind MHC class I molecules, namely HLA-E and possibly HLA-G [33,34]. Expression of transgenic HLA-E and HLA-G in endothelial cell lines was reported to significantly suppress macrophage-mediated cytotoxicity in a xenomodel [35,36]. Furthermore, expression of HLA-E and HLA-G was associated with a reduced rate of rejection in transplant recipients [37,38,39]. CD161 is primarily an inhibitory receptor, blockage of which promotes activation of T cells and cytotoxicity [40,41]. CD161 is a marker of pro-inflammatory NK cell function with high cytokine responsiveness [42]. CD161+ T cells present an important subset of early inflammatory cells in allograft rejection, but their relative contribution and significance compared to other immune cells remain to be explored [43,44]. Granzyme B, which is encoded by the GZMB gene, was shown to be significantly overexpressed in patients with acute solid organ rejection compared to stable patients. Interestingly, a significant decrease in expression levels of this enzyme was noted after initiation of anti-rejection therapy [27,28,29]. Granzyme B plays a key role in inducing apoptosis in target cells during immune responses and mediating early allograft injury [45,46]. Several recent studies investigated the possibilities of its application for noninvasive diagnosis of transplant rejection [30,31]. Finally, GNLY (cytolytic protein expressed in NK cells) and PLA1A (phospholipase A1 member A, an IFNG-inducible enzyme) are antibody-mediated selective transcripts [26,32]. Increased expression of these two genes was observed in rejecting human hearts [47,48]. GNLY (granulysin) contributes to tissue damage and allograft rejection by promoting cytotoxicity and inflammation [49]. In addition, granulysin can induce targeted allograft apoptosis through perforin-dependent and perforin-independent mechanisms [50,51]. Granulysin binds to phospholipids in cell membranes, which is important for its cytotoxic function as it allows the protein to disrupt the integrity of cell membranes and induce cell death [52,53]. The specific role of PLA1A (IFNG-inducible enzyme) in rejection mechanisms is less clear, but phospholipases can modulate inflammatory responses and immune cell functions by generating lipid mediators [54]. An increase in the activity of phospholipases triggers lipid degradation and subsequently energy metabolism imbalance [55,56]. In order to predict acute allograft rejection based on gene expression profiles, we trained eight machine learning algorithms to identify the best-performing one. Machine learning models are at high risk of overfitting when trained on datasets with a large number of features relative to the number of observations [57,58]. Overfitting occurs when a model learns the noise in the training data to the extent that it performs poorly on new, unseen data. It is a common issue in bioinformatics analyses of gene expression datasets obtained from publicly available repositories such as GEO [59]. In our study, the GSE150059 dataset that was used to train the models contains hundreds of samples and is therefore large enough for this task. In addition, we ensured that our models were not overfitting through rigorous feature selection to reduce dimensionality, cross-validation, and internal validation. Various metrics were used to assess the predictive performance of each model as it is impossible for any single metric to capture all the strengths and weaknesses of a classifier, especially in the setting of an unbalanced dataset or in the presence of confounders [19]. For instance, if we had used only F1 score (aggregate metric) or AUPRC, we would have mistakenly assumed that some models had good performance in predicting histologic diagnosis of acute cardiac rejection.

We achieved excellent predictive performance in all the machine learning algorithms trained on these six genes with RF and LR outperforming the other six models. In addition, we ran each model on two external validation sets. However, this was conducted to assess differences between MMDx and histologic diagnoses rather than evaluate model performance. Despite great predictive performance shown in test and validation sets, all the models failed to achieve optimal results in two external validation sets. Thus, our study highlights major differences between the two diagnostic methods in terms of machine learning algorithms. Discrepancies between histologic and MMDx diagnosis in solid organ rejection were reported in multiple studies [60,61,62]. Although MMDx cannot yet replace histopathology, both methods complement each other and help expand our understanding of heart transplant rejection states [63]. Apart from differences in diagnostic modalities, the performance of machine learning algorithms was likely affected by several factors, albeit to a smaller degree. Firstly, only normalized data of both external validation sets could be obtained from the GEO. Utilization of different normalization methods is known to affect model performance [64]. Secondly, the first external validation set had missing values, which were replaced with a median. Thirdly, only genes that were present in all the datasets were selected.

LIME and SHAP are two techniques used to explain the predictions made by machine learning models. LIME focuses on generating local, interpretable explanations for individual predictions. It does this by approximating the behavior of the model around a specific prediction using a simple, interpretable model. This involves sampling perturbations around the prediction and fitting a straightforward model to these perturbations. By doing so, LIME can identify which features have the most significant influence on the prediction [65]. In contrast, SHAP provides a more global explanation by utilizing Shapley values from cooperative game theory to measure the contribution of each feature to the prediction. It achieves this by estimating the marginal contribution of each feature through an iterative process of adding features to a reference value and observing the resulting change in the model output. The resulting feature attributions offer an additive explanation of how each feature contributes to the overall model output [66]. Both LIME and SHAP offer valuable insights into the contributions of different features towards model predictions. These insights can be particularly useful in understanding the mechanisms involved in complex phenomena such as cardiac rejection. By gaining a better understanding of feature importance, researchers can potentially develop preventive or therapeutic interventions. HCP5, KLRD1, GZMB, PLA1A, GNLY, and KLRB1 emerged as robust biomarkers for molecular diagnosis of acute cardiac rejection and had a prediction probability of 1.0. KLRD1 and HCP5 were identified as the most impactful by SHAP and LIME, highlighting their importance in cardiac rejection and potential as therapeutic targets. In the future, therapeutic interventions may be tailored to individual transplant recipients based on their unique gene expression profiles. For instance, high-risk patients identified by the predictive model may be closely monitored for signs of rejection and receive appropriate treatment, while those at low risk may require less aggressive immunosuppressive therapy, thus reducing the risk of adverse effects.

## 5. Conclusions

Taken together, machine learning algorithms hold immense promise for advancing therapeutic and preventive strategies in acute cardiac allograft rejection. LR, SVM, RF, GBM, KNN, XGBoost, DT, and neutral networks demonstrated great predictive performance in predicting acute cardiac rejection based on MMDx. LR and RF outperformed the other six machine learning models. However, all models showed poor performance when predicting histologic diagnosis of cardiac rejection, which is attributed to differences between these two methods. HCP5, KLRD1, GZMB, PLA1A, GNLY, and KLRB1 were identified as candidate genes. According to SHAP and LIME, KLRD1 and HCP5 were the most impactful genes.

## Figures and Tables

**Table 1:** Datasets used in this study.

Dataset	Number of Acute Cardiac Rejection Samples	Number of Non-Rejection Samples	Platform	Rejection Diagnosis	Set
GSE150059	853	467	GPL16043	MMDx	Training set, test set, internal validation set
GSE2596	35	21	GPL1053	Histology	External validation set 1
GSE4470	15	12	GPL1053	Histology	External validation set 1
GSE9377	17	9	GPL887	Histology	External validation set 2

**Table 2:** Results of machine learning algorithms trained on six genes (HCP5, KLRD1, GZMB, PLA1A, GNLY, and KLRB1) in test and internal validation sets.

Metric	RF	LR	DT	SVM	GBM	KNN	XGB	MLP
Test set (MMDx)								
Accuracy	0.95	0.95	0.91	0.93	0.92	0.93	0.94	0.93
Precision	0.95	0.95	0.92	0.93	0.92	0.93	0.95	0.90
Recall	0.90	0.90	0.81	0.89	0.86	0.89	0.89	0.90
F1 Score	0.93	0.93	0.86	0.91	0.89	0.91	0.92	0.90
AUC	0.98	0.98	0.90	0.98	0.98	0.98	0.98	0.98
MCC	0.89	0.89	0.80	0.86	0.83	0.86	0.88	0.85
AUPRC	0.97	0.98	0.90	0.97	0.97	0.97	0.97	0.97
Internal validation set (MMDx)								
Accuracy	0.89	0.90	0.87	0.90	0.91	0.90	0.90	0.89
Precision	0.87	0.88	0.84	0.88	0.89	0.88	0.88	0.84
Recall	0.83	0.83	0.80	0.83	0.84	0.83	0.83	0.84
F1 Score	0.85	0.85	0.82	0.85	0.87	0.85	0.85	0.84
AUC	0.96	0.96	0.88	0.96	0.96	0.94	0.95	0.96
MCC	0.77	0.78	0.72	0.78	0.80	0.78	0.78	0.76
AUPRC	0.93	0.92	0.86	0.91	0.92	0.90	0.90	0.92

Note: RF—random forest, LR—logistic regression, DT—decision trees, SVM—support vector machines, GBM—gradient boosting machines, KNN—K-nearest neighbors, XGB—XGBoost, MLP—multilayer perception (neural network), AUC—area under the curve, MCC—Matthew’s correlation coefficient, AUPRC—area under the precision–recall curve.

**Table 3:** Results of machine learning algorithms trained on six genes (HCP5, KLRD1, GZMB, PLA1A, GNLY, and KLRB1) in two external validation sets.

Metric	RF	LR	DT	SVM	GBM	KNN	XGB	MLP
External validation set 1 (histology)								
Accuracy	0.46	0.45	0.48	0.46	0.42	0.41	0.46	0.45
Precision	0.42	0.42	0.43	0.42	0.4	0.39	0.4	0.42
Recall	0.97	1	0.91	1	0.88	0.85	0.73	1
F1 Score	0.59	0.59	0.58	0.59	0.55	0.53	0.52	0.59
AUC	0.55	0.48	0.57	0.57	0.53	0.52	0.51	0.47
MCC	0.16	0.18	0.15	0.21	0	−0.05	0.01	0.18
AUPRC	0.45	0.35	0.66	0.69	0.4	0.6	0.38	0.35
External validation set 2 (histology)								
Accuracy	0.65	0.54	0.27	0.35	0.69	0.54	0.42	0.54
Precision	0.75	0.73	0.33	0	0.8	0.73	0.62	0.73
Recall	0.71	0.47	0.12	0	0.71	0.47	0.29	0.47
F1 Score	0.73	0.57	0.17	0	0.75	0.57	0.4	0.57
AUC	0.5	0.49	0.27	0.5	0.56	0.52	0.52	0.48
MCC	0.26	0.13	−0.37	0	0.36	0.13	−0.04	0.13
AUPRC	0.67	0.66	0.53	0.66	0.7	0.66	0.72	0.65

Note: External validation set 1—merged GSE2596 and GSE4470, external validation set 2—GSE9377, RF—random forest, LR—logistic regression, DT—decision trees, SVM—support vector machines, GBM—gradient boosting machines, KNN—K-nearest neighbors, XGB—XGBoost, MLP—multilayer perception (neural network), AUC—area under the curve, MCC—Matthew’s correlation coefficient, AUPRC—area under the precision–recall curve.

## Data Availability

The data presented in this study are available in the Gene Expression Omnibus at https://www.ncbi.nlm.nih.gov/geo/query/acc.cgi?acc=GSE150059, https://www.ncbi.nlm.nih.gov/geo/query/acc.cgi?acc=GSE9377, https://www.ncbi.nlm.nih.gov/geo/query/acc.cgi?acc=GSE2596, and https://www.ncbi.nlm.nih.gov/geo/query/acc.cgi?acc=GSE4470 all accessed on accessed on 24 March 2024.
